# Acceptability and Feasibility of a Return-to-Work Intervention for Posttreatment Breast Cancer Survivors: Protocol for a Co-design and Development Study

**DOI:** 10.2196/37009

**Published:** 2022-04-22

**Authors:** Karine Bilodeau, Marie-Michelle Gouin, Alexandra Lecours, Valérie Lederer, Marie-José Durand, Kelley Kilpatrick, David Lepage, Lauriane Ladouceur-Deslauriers, Tomas Dorta

**Affiliations:** 1 Faculty of Nursing University of Montreal Montreal, QC Canada; 2 Centre de recherche Hopital Maisonneuve Rosemont Montreal, QC Canada; 3 Faculté de médecine et des sciences de la santé University of Sherbrooke Longueuil, QC Canada; 4 Département de relations industrielles Université du Québec à Trois-Rivières Trois-Rivières, QC Canada; 5 Département de relations industrielles Université du Québec en Outaouais Gatineau, QC Canada; 6 Ingram School of Nursing Mcgill University Montreal, QC Canada; 7 Centre intégré universitaire de santé et de services sociaux de l'Est de l'île de Montréal Montréal, QC Canada; 8 Faculté de l'aménagement École de Design Université de Montréal Montreal, QC Canada

**Keywords:** co-design, breast cancer, intervention, return-to-work, primary care, qualitative

## Abstract

**Background:**

The mortality rate from breast cancer has been declining for many years, and the population size of working-age survivors is steadily increasing. However, the recurrent side effects of cancer and its treatment can result in multiple disabilities and disruptions to day-to-day life, including work disruptions. Despite the existing knowledge of best practices regarding return to work (RTW) for breast cancer survivors, only a few interdisciplinary interventions have been developed to address the individualized needs and multiple challenges of breast cancer survivors, health care professionals, and employer and insurer representatives. Thus, it seems appropriate to develop RTW interventions collaboratively by using a co-design approach with these specific stakeholders.

**Objective:**

This paper presents a protocol for developing and testing an innovative, interdisciplinary pilot intervention based on a co-design approach to better support RTW and job retention after breast cancer treatment.

**Methods:**

First, a participatory research approach will be used to develop the intervention in a co-design workshop with 12 to 20 participants, including people affected by cancer, employer and insurer representatives, and health care professionals. Next, a pilot intervention will be tested in a primary care setting with 6 to 8 women affected by breast cancer. The acceptability and feasibility of the pilot intervention will be pretested through semistructured interviews with participants, health care professionals, and involved patient partners. The transcribed data will undergo an iterative content analysis.

**Results:**

The first phase of the project—the co-design workshop—was completed in June 2021. The pilot test of the intervention will begin in spring 2022. The results from the test will be available in late 2022.

**Conclusions:**

The project will offer novel data regarding the use of the co-design approach for the development of innovative, co-designed interventions. In addition, it will be possible to document the acceptability and feasibility of the pilot intervention with a primary care team. Depending on the results obtained, the intervention could be implemented on a larger scale.

**International Registered Report Identifier (IRRID):**

DERR1-10.2196/37009

## Introduction

It has been reported that 1 in 8 Canadian women will develop breast cancer in their lifetime [1,2]. Of these, 88% will survive for more than 5 years, and of these survivors, 50% are of working age. In the current context of labor shortages [3], the contribution of those with a cancer diagnosis who want to return to work (RTW) is valuable. Indeed, many survivors desire to RTW because it is a sign of a return to a “normal” life [2,4-7]. On the other hand, 1 in 5 women will leave their jobs due to an inability to perform their tasks resulting from recurrent side effects of cancer or its treatments [8]. There are a variety of reasons for the development of these limiting side effects. Breast cancer treatments, such as mastectomy or chemotherapy, can cause significant physical symptoms [9] (eg, pain, the loss of arm mobility, and lymphedema) that may limit the act of lifting or work involving repetitive motions [10]. These symptoms and physical limitations can severely complicate and even hinder daily work [7,10]. Fatigue, as well as memory loss and difficulty concentrating, can also impact RTW [11]. Furthermore, breast cancer survivors often live more precariously than the general population due to taking repeated sick leaves from work or having only part-time employment after cancer treatments [12-16]. Despite these challenges, both working and having good working conditions are related to better health [17], as is allowing for the maintenance of social interactions, self-esteem, psychological well-being, and financial security [2,18,19].

Interdisciplinary interventions that support RTW and are specifically tailored to the issues experienced by breast cancer survivors remain rare in Canada and do not exist in Quebec, and this scarcity of tailored interventions seems likely to continue [20-22]. Indeed, a Cochrane review about RTW interventions for patients who have completed cancer treatments revealed that interventions that targeted multiple modalities (physical, psychological, and vocational) and were delivered by an interdisciplinary team seemed more appropriate for addressing the needs of cancer survivors [23]. This multimodal type of intervention appears promising, although the effect size remained tenuous and suggested the need for further study [23]. Other studies suggest using more comprehensive approaches, such as discussing work-related issues during psycho-oncological care [24,25], involving employers [26], and performing early intervention [27]. It has even been suggested that work-related issues should be discussed as early as the active treatment period to maintain social roles, including those of workers [25,28]. According to a scoping review [27], RTW interventions that have been identified to support breast cancer survivors are offered in ad hoc interventions by different health care professionals and are highly variable in terms of information booklets, physical activities, and deployment times (eg, at the end of treatment) [27]. More structured interdisciplinary interventions remain to be developed to meet the unique needs of this clientele regarding RTW.

It seems desirable and realistic that interdisciplinary interventions be offered by a primary care team [20]. By definition, primary care can include health promotion, disease prevention, the monitoring of chronic and episodic diseases, and rehabilitation [29]. It is also possible that such a team would be able to address the RTW-related concerns of women affected by breast cancer, which include symptom management, RTW-related decision-making, resource navigation, and the reintegration of daily activities [4,30]. Primary care teams promote interdisciplinary work and provide services as close to a given population as possible [31]. Furthermore, given the scope of primary care, it is strongly recommended that those affected by cancer be managed by such teams during the recovery period [32,33]. It is also suggested that intervention components should be deployed during key points in the experience of cancer survivorship at 1, 3, and 6 months after cancer treatment and encourage the self-management of side effects (eg, cognitive difficulties and fatigue), RTW-related decision-making, resource navigation, and the reintegration of daily activities [4,21]. As for the intensity of interventions that should be offered, it is mentioned that nearly 50% of affected individuals require more assiduous support due to the presence of persistent side effects [34], such as severe fatigue or physical limitations induced by lymphedema [35,36]. Moreover, studies have shown that women affected by breast cancer who have received chemotherapy treatments experience more difficulty during RTW due to persistent side effects [37-40]. This clientele therefore should receive personalized support for coping with the challenges of completing cancer treatments, including RTW.

RTW after cancer treatment is complex because it involves multiple stakeholders [41], including breast cancer survivors, health care professionals, employers, and insurer representatives, who come from multiple settings and have divergent concerns, constraints, and resources. The available interventions that support RTW after cancer treatment have proven to be insufficient in connecting workplace stakeholders and addressing RTW-related coordination challenges [20,27]. Our project aims to develop a supportive RTW intervention to address the current lack of coordinated, interdisciplinary interventions offered in primary care. Based on current evidence, it is imperative to develop new interventions to support the RTW of breast cancer survivors. These interventions should have several characteristics that align with the Medical Research Council criteria [42]. As such, these interventions will be complex, especially given the variety of the intervention components needed, the interactions between these components, the number of people or organizational levels involved, the degree of flexibility required, and the need for the ability to adapt to specific contexts. The development of a complex intervention requires several phases that do not follow a linear sequence [42]. To address this complexity, it is advisable to combine theoretical, empirical, and experiential approaches when designing interventions [43]. However, these approaches are time-consuming, and it is difficult to address all of the issues related to implementing interventions [44]. To engage stakeholders and achieve contextually appropriate intervention components, our project will involve a coideation process for developing an RTW intervention and its components through a co-design approach. This novel methodological approach to developing RTW interventions brings together cross-sectoral stakeholders, including health care professionals, employer and insurer representatives, and breast cancer survivors. The components of interventions can be difficult for stakeholders to understand, and such components can be difficult to contextualize during the development process. The co-design approach is therefore appropriate, as it was developed so that stakeholders can actively participate in a process of coideation for the interventions that are dedicated to them. The proposed approach differs from others because it offers the possibility for all participants to cocreate and negotiate ideas simultaneously, starting from the beginning (conceptual phase) of the co-design process [45]. Another characteristic of the approach is the presence of design professionals who facilitate the coideation process [46] by making graphic representations that are adapted to the participants to support the creative process. Finally, coideation has proven to be an innovative approach that has several potential benefits. First, coideation allows for the creation of an intervention without preconceived ideas. Second, the creation of an intervention can be faster, allowing for quicker testing in a pilot study context. This provides better direction for subsequent developments.

Given the need for support among breast cancer survivors, the inherent challenges of RTW, and the complexity of intervening to support RTW, there is a need to coconstruct a coordinated, interdisciplinary intervention with RTW stakeholders that is offered by a primary care team. To achieve this, we propose a co-design approach. The goal of the study is to develop and test a pilot intervention for supporting RTW after breast cancer treatment. The intervention will be offered by a primary care team. The objectives are to (1) develop an innovative intervention in collaboration with key stakeholders, (2) test the intervention with a primary care team, and (3) determine the acceptability and feasibility of the intervention in a primary care setting.

## Methods

**Figure 1:**
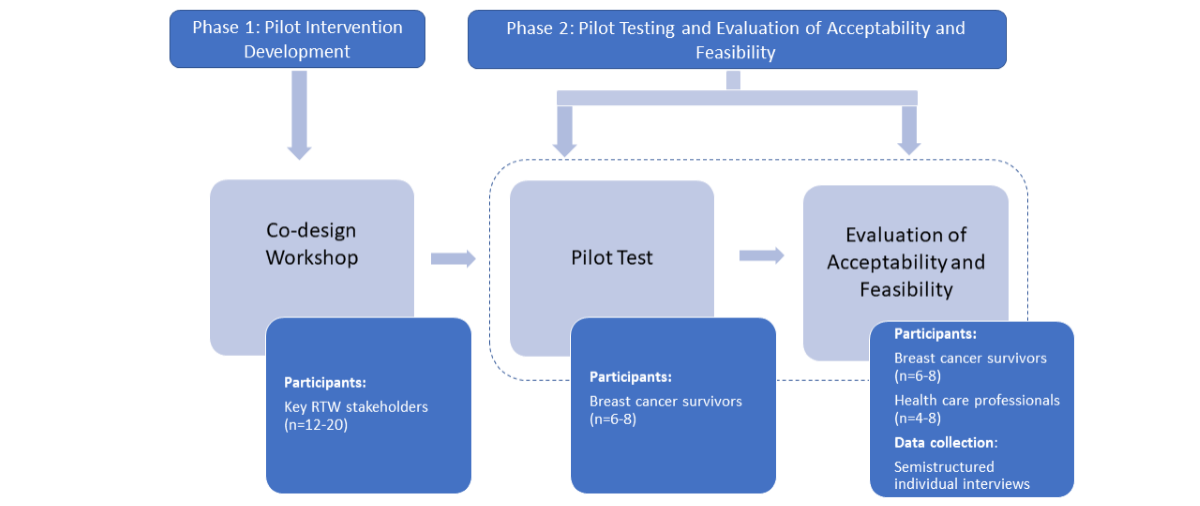
Study design. RTW: return to work.

The project is divided into the following two phases: the development of the pilot intervention and the feasibility assessment (Figure 1).

### Phase 1: Pilot Intervention Development

#### Study Design

A participatory action research design [47] and a co-design approach [48] will be used. A participatory action design engages researchers and participants in a reflective process when they seek solutions [47,49]. In addition, it encourages the partnership between researchers and stakeholders throughout the research process. Participatory research makes it possible to produce useful and precise knowledge that is consistent with the realities of key RTW stakeholders. The synergy created in partnerships allows for studies that are both culturally appropriate for target audiences and logistically realistic [50]. The development of the pilot intervention will be inspired by the reflexive cycle—a key part of the participatory approach that encourages describing and defining observations, analyzing and interpretating ideas, and finding solutions [49,51]. In our case, the solutions will be creative and innovative. This process will take place during a co-design workshop with key stakeholders. Co-design allows people to actively participate in a process of coideation for finding innovative solutions, during which they can simultaneously participate in the cocreation process at the beginning of a project by following a democratic and multidisciplinary perspective [48].

#### Population and Recruitment

A variety of participants (n=12-20) representing key RTW stakeholders will participate in the codevelopment of the pilot intervention. For this phase, snowball sampling will be conducted [52]. All participants must speak French and be aged ≥18 years. To be selected for the codevelopment phase, breast cancer survivors must have experienced RTW after cancer treatments for at least 1 year, employer or insurer representatives must have at least coordinated or accompanied the RTW of a woman affected by breast cancer, and health care professionals must have previously provided support to a woman with breast cancer during either oncology or primary care. Participants will be approached through the research team's professional networks. This strategy is appropriate, given the nature of the interactive activity and the need for participants to be engaged and involved in the RTW journey. These are features of some participatory and co-design approaches [53].

#### Sequence of the Co-design Workshop

In preparation for the workshop, a 10-minute informational video will be sent to participants. This video will present the current knowledge regarding RTW for cancer survivors as well as recommendations from research. By using the Zoom platform (Zoom Video Communications Inc), a co-design workshop will be held over 4 hours. Participants will be informed that the purpose of the workshop is to co-design components of a pilot intervention that will be performed by a primary care team to address the issues faced by survivors who RTW after breast cancer treatment. Further, 3 case scenarios that represent the potential issues of RTW after breast cancer treatment will be presented to participants. Because the workshop will include key RTW stakeholders, the scenarios will serve as a “representative artifact,” that is, a deliberation strategy involving interactions between public participants and health professionals that encourage exchanges via a common and appropriate language [54]. Participants will also be guided through the following two dimensions of intervention: the assessment of cancer and treatment side effects and RTW discussion and planning. Participants will be instructed to not work on existing or unappreciated resources for people affected by cancer, such as a pamphlet or a website that already exists in Canada [55]. Instead, participants will be invited to think of novel solutions.

**Figure 2:**
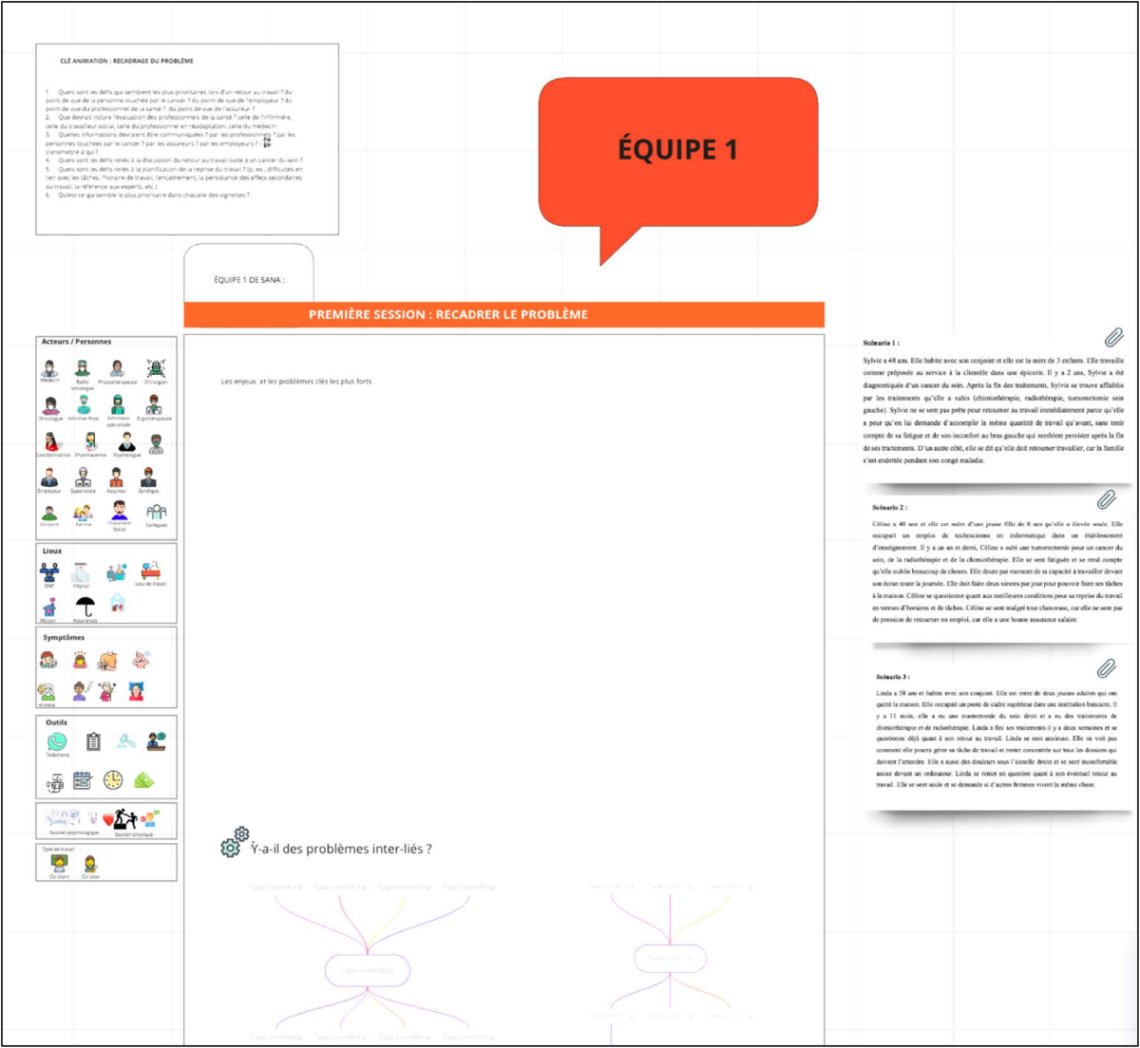
Miro interface for “Reframing the problem.”

**Figure 3:**
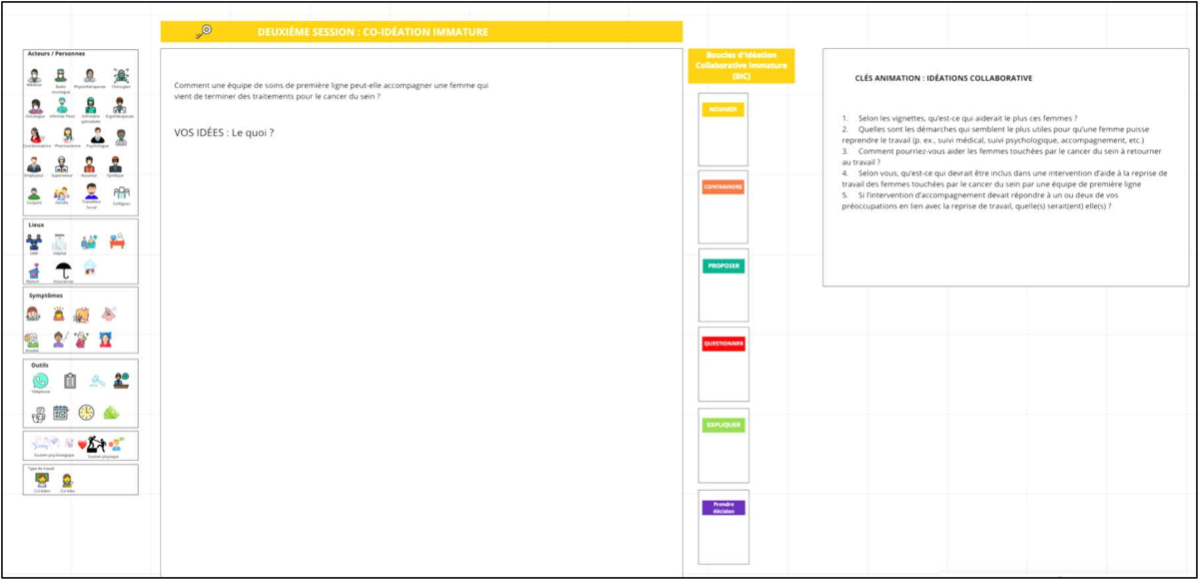
Miro interface for “Immature coideation.”

**Figure 4:**
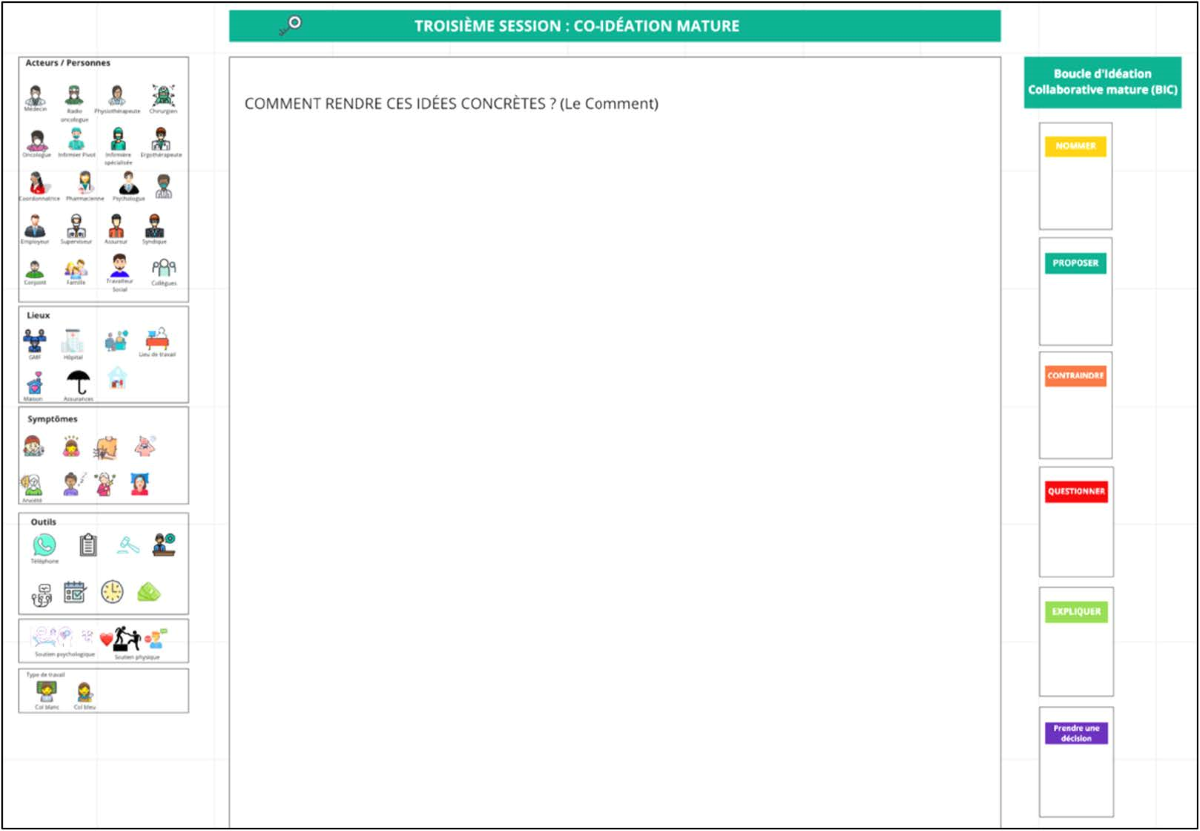
Miro interface for “Mature coideation.”

During the workshop, participants will interact in plenary and breakout sessions. Further, 2 to 4 diverse subgroups will be created, depending on the number of participants and the representativeness of the RTW stakeholders, for activities in a breakout room. Each subgroup will be accompanied by a facilitator and a design professional who will help to frame the discussions. The facilitator will be a postdoctoral fellow or a graduate student from an undergraduate design program (eg, industrial design). The involvement of design professionals is recommended for health science initiatives [46]. As presented in Table 1, the workshop will be divided into the following five steps: (1) the reframing of the problem, (2) immature coideation, (3) mature coideation, (4) the presentation of subgroup solutions, and (5) the debriefing of the workshop experience. In step 1, participants will be invited to discuss the problem of RTW after cancer treatment. The facilitator can refer to the case scenarios and frame the discussions to raise priority issues. In step 2, participants will be asked to develop solutions. The facilitator will assist the participants in developing solutions that are relevant to issues that were identified earlier and representing them graphically (eg, diagrams and drawings). At this stage, the ideas are to be formulated but not yet fully developed. In step 3, participants will be invited to fully develop the solutions that they find the most important, in some cases by making more detailed graphic representations. At this stage, specific details will be offered by participants in each subgroup with regard to the implementation or components of the selected solutions. In step 4, a plenary discussion will be held to summarize the work of each subgroup. Finally, in step 5, the workshop will end with a debriefing on the participants' perceptions of the positive and negative aspects to be retained or considered for the repetition of the workshop. Of note, throughout the workshop, participants will make use of a graphical representational ecosystem (eg, drawings and digital sketches) [56], along with the verbal exchanges, to externalize their ideas and discuss proposals more clearly. In addition, subgroup discussions can be structured by using the following key principles of design conversation [48,57]: (1) naming the problem, (2) constraining the ideas, (3) proposing the ideas (the key element), (4) negotiating the ideas through questioning and explaining, (5) making decisions, and (6) making design advances with graphical representations. This approach keeps discussions focused on a common goal and ensures that stakeholders collaborate within a limited time frame during all of the workshop stages. The activity will take place on the internet due to the current pandemic context and will be conducted by using the Miro platform (Participatory Culture Foundation; Figures 2-4). A pretest of the workshop will be conducted with students before the activity.

The workshop will be recorded, and explicit notes will be written verbatim. The deliverable at this stage will be the general guidelines for an intervention that supports RTW after breast cancer treatment. The intervention will be designed so that it is fit for use in primary care settings.

**Table 1:** Sequence of the co-design workshop.

Activities and steps		Length (total: 235 minutes)
**Welcome and introduction**		10 minutes (plenary session)
	Step 1: reframing the problem	30 minutes (subgroup session) 15 minutes (plenary session)
**Transition and breakout room integration**		5 minutes
	Step 2: immature coideation	30 minutes (subgroup session) 15 minutes (plenary session)
**Break**		15 minutes
	Step 3: mature coideation	30 minutes (subgroup session) 15 minutes (plenary session)
**Transition and breakout room integration**		5 minutes
	Step 4: presentation of subgroup solutions	30 minutes (plenary session)
**Transition and breakout room integration**		5 minutes
	Step 5: debriefing the workshop experience	30 minutes (plenary session)

### Phase 2: Pilot Test and Evaluation of Acceptability and Feasibility

The pilot intervention will be delivered by a primary care team and patient partners who will be trained in the intervention. A resource person will be available to answer their questions or provide coaching during the project.

#### Population and Recruitment

By using purposive sampling [58], 6 to 8 breast cancer survivors will be recruited to test the pilot intervention. The selection criteria will include women aged 18 to 60 years; those who have completed breast cancer treatments, such as surgery, chemotherapy (doxorubicin, cyclophosphamide, and paclitaxel), and radiation therapy; and those who are planning to RTW within the next year. Surgeon oncologists will help with targeting potential participants who are nearing the completion of their cancer treatments.

#### Evaluation of Acceptability and Feasibility

At the end of the project, the acceptability and feasibility of the pilot intervention will be measured by conducting semistructured individual interviews with participants (n=6-8) and health care professionals, as well as patient partners who participated in the intervention (n=4-8). The use of qualitative interviews is recommended during a feasibility study [59]. The interview guide and analysis will be based on the Theoretical Framework of Acceptability [59], which explains that acceptability is a multidimensional construct with 7 components. Specifically, the interview questions will address (1) affective attitudes toward the intervention, (2) the effort required to complete the intervention, (3) consistency with the individuals’ values, (4) individuals’ understanding of the intervention and how it works, (5) the perceived benefits provided by the intervention, (6) perceived efficacy, and (7) the perceived ability to complete the intervention. The interview guide will be pretested with 2 people who have the same characteristics as those of the participants.

#### Data Analysis

The transcribed interview will undergo an iterative content analysis, which will include the following activities: condensation, data presentation, and the development and verification of findings [60]. Further, 2 team members will conduct the coding process. The data from the breast cancer survivors will be contrasted to highlight similarities and differences in experiences with the pilot intervention. The same exercise will be conducted for the health care professionals. In addition, to validate the emerging findings, a discussion with the coresearchers will be conducted throughout the research process. This discussion will consider the data transcripts and field notes. NVivo software (QSR International) will be used for qualitative data management. To ensure the quality of the study, techniques such as data triangulation will be used to ensure internal credibility and validity. Further, we will validate some of the participants’ data to ensure their reliability, assess the procedural documentation of the research process to determine accountability, and provide a detailed description of the context to ensure external transferability and validity [60].

### Ethics Approval

Ethics approval was granted in May 2021 for the first component of the project (Centre intégré universitaire de santé et de services sociaux de l'Est-de-l'Île-de-Montréal; project number: #2022-2610).

## Results

The project received funding in March 2021. The co-design workshop took place on June 16, 2021. A total of 11 people participated in the activity. The transcripts of the discussions were analyzed and helped to target the following intervention themes for intervention development: (1) mitigating the assessment and self-management of side effects, (2) assessing RTW needs and abilities, and (3) communicating with the employer. The clinical tools for the intervention (questionnaire and decision support tree) and the intervention logic model remain to be finalized. The preliminary results suggest that the pilot intervention should take place in a primary care setting in the Montreal area (Quebec, Canada). It is anticipated that 3 meetings will occur at 1, 4, and 6 months after treatment is completed [21]. During these meetings, a health professional will analyze RTW challenges and may propose solutions that are tailored to women’s needs. The intervention will include pairing participants with patient partners who will be able to answer the participants’ questions and share their RTW experiences [61]. Depending on the pandemic context of COVID-19 in Canada, recruitment will begin in spring 2022.

## Discussion

### Principal Results

In brief, the project will allow us to document the relevance of an RTW intervention that is delivered by a primary care team. The shared views of patients and health care professionals will help us determine the feasibility and acceptability of the intervention. In addition, the project will offer methodological recommendations for the use of a co-design approach in intervention development. More specifically, the project will have clinical, methodological, and organizational benefits.

On the clinical level, the primary outcome of the project is the development of a program that supports RTW and is based on the perspectives of RTW stakeholders, including breast cancer survivors [41]. Only a few European studies have mobilized these actors for the development of RTW interventions for patients who have completed cancer treatments [62,63]. The participatory approach that will be used in the project properly contextualizes the intervention to a Canadian context. This should facilitate implementation in the second phase of the study. The results of the pilot project will also provide information for improving the intervention before large-scale implementation, which will make it possible to evaluate its effects. The project will also offer an initial solution for women affected by breast cancer who need support [64] but are not covered by current health services [21]. Indeed, it is more urgent than ever to address the issues of RTW after cancer treatment to limit the development of disabilities, particularly as work is a known determinant of health and social participation is beneficial to individuals [17,64]. Additionally, and perhaps more broadly, RTW-related disabilities result in considerable consequences that also affect society as a whole [65-67], especially when absences are prolonged over time [65]. Fostering RTW is therefore essential, especially in the context of an aging population and labor shortages [68].

In terms of methodology, the project will explore an innovative and participatory approach that is perfectly suited to the trend of including patients and those who are involved in creating interventions (eg, a patient-centered research strategy [69]). To engage patients and partners, the use of approaches that have been adapted from design-related approaches [46] (eg, experience-based design [70] and Hacking Health hackathons [71]) has become increasingly common in health care. These approaches are appreciated and are essential, given the complexity of the interventions that are to be developed in health care [42]. That said, many deplore the time and investment required to complete such processes [72,73]. Our project addresses this concern via the use of a structured co-design ideation approach to develop innovative and robust solutions within a reasonable time frame. Through this approach, we will be able to decrease intervention development time, increase cross-sectoral stakeholder participation, and make the intervention creation process more efficient. The coideation process will promote the creation of robust solutions for effective implementation and result in a decrease in the time required for the intervention creation cycle (ie, from ideation to implementation). The new data provided by our project will facilitate the development of new interdisciplinary interventions that benefit clientele in various health care settings.

This protocol is the first to propose a pilot RTW intervention for patients who have completed cancer treatments that will be delivered by a primary care team. It is widely documented that the supply of care after cancer treatment is inadequate [21,74,75]. It remains difficult to provide services to people affected by cancer beyond specialized oncology services, despite numerous international recommendations [76]. It is hoped that our RTW-themed project will encourage health care professionals and employers to commit to its goals and principles by demonstrating that the intervention will be acceptable to participants and consistent with the mission of primary care teams (ie, promoting health and preventing disease). The project will thus offer new data on the feasibility of offering this follow-up intervention in a primary care setting.

### Anticipated Challenges and Limitations

This protocol is proposed in a Canadian context (province of Quebec). In Canada, health care is free and primary care services are readily accessible to the general population. It should be noted that the majority of Canadians affected by cancer have paid sick leave included in their private insurance contracts [16]. Many people, including those without private insurance, only have access to 15 weeks of compensation from the Canadian government. Moreover, with regard to RTW after cancer treatment, no agreement has been reached with employers, and no legislation has been created, as is the case in France. This protocol must therefore be interpreted in this context. Furthermore, the evaluation of feasibility and acceptability will be based on a few qualitative interviews. The number of interviews should be sufficient, that is, from a scientific point of view [77], for documenting whether the intervention is feasible in this context. Finally, the pandemic context of COVID-19 may limit the testing of the project. Primary care teams are facing the off-loading of their professionals to other areas, including those that require vaccination efforts. To mitigate these effects, we will rely on the support of primary care physicians; oncology specialists; and members of the research team (DL and LLD), who are also clinicians. Despite the unfavorable context, health care professionals have reiterated their willingness to move forward, which demonstrates the relevance of the project and the interest of the health care organizations.

### Conclusions

This protocol proposes to develop and test an intervention that supports RTW after breast cancer treatment and is delivered by a primary care team. The project will provide novel data on the use of a co-design method for the development of complex interventions. In addition, the results of the project will allow us to better document the acceptability and feasibility of the intervention, which will be delivered by a primary care team. Depending on the results obtained, the project could be tested on a larger scale.

## Appendix

Multimedia Appendix 1 Peer-review reports.

## Abbreviations

RTW: return to work
