# Correction: Acceptability and Feasibility of a Return-to-Work Intervention for Posttreatment Breast Cancer Survivors: Protocol for a Co-design and Development Study

**DOI:** 10.2196/50784

**Published:** 2023-07-19

**Authors:** Karine Bilodeau, Marie-Michelle Gouin, Alexandra Lecours, Valérie Lederer, Marie-José Durand, Kelley Kilpatrick, David Lepage, Lauriane Ladouceur-Deslauriers, Tomas Dorta

**Affiliations:** 1 Faculty of Nursing University of Montreal Montreal, QC Canada; 2 Centre de recherche Hopital Maisonneuve Rosemont Montreal, QC Canada; 3 Faculté de médecine et des sciences de la santé University of Sherbrooke Longueuil, QC Canada; 4 Département de relations industrielles Université du Québec à Trois-Rivières Trois-Rivières, QC Canada; 5 Département de relations industrielles Université du Québec en Outaouais Gatineau, QC Canada; 6 Ingram School of Nursing Mcgill University Montreal, QC Canada; 7 Centre intégré universitaire de santé et de services sociaux de l'Est de l'île de Montréal Montréal, QC Canada; 8 Faculté de l'aménagement École de Design Université de Montréal Montreal, QC Canada

In “Acceptability and Feasibility of a Return-to-Work Intervention for Posttreatment Breast Cancer Survivors: Protocol for a Co-design and Development Study” (JMIR Res Protoc 2022;11(4):e37009) the authors noted an error.

The following sentence and reference [55] were deleted from the manuscript:

Participants will be instructed to not work on existing or unappreciated resources for people affected by cancer, such as a pamphlet or a website that already exists in Canada [55].

All subsequent references have been renumbered accordingly.

The correction will appear in the online version of the paper on the JMIR Publications website on July 18, 2023, together with the publication of this correction notice. Because this was made after submission to PubMed, PubMed Central, and other full-text repositories, the corrected article has also been resubmitted to those repositories.
