# Supplementary material for: Acceptability and Feasibility of a Return-to-Work Intervention for Posttreatment Breast Cancer Survivors: Protocol for a Co-design and Development Study
Source: JMIR Res Protoc. 2022 Apr 22;11(4):e37009. doi: 10.2196/37009 (PMC9077508; doi:10.2196/37009)
Supplement: Multimedia Appendix 1 [file resprot_v11i4e37009_app1.pdf]

REPAR

Réseau thématique soutenu par le FRQS

Montréal, le 15 février 2021

Madame Karine Bilodeau *Par courriel*
karine.bilodeau.2@umontreal.ca

Objet : Votre demande de subvention au Programme 1.1 Recherche clinique 2020-2021

Titre du projet : « Développer et mettre à l'essai une intervention interdisciplinaire pour soutenir la reprise du travail de survivantes du cancer du sein par une équipe en première ligne »

Codemandeurs **:** *Marie-Josée Durand, Marie-Michelle Gouin, Alexandra Lecours, Valérie Lederer, Kelley Kilpatrick, David Lepage, Lauriane Ladouceur-Deslauriers*

Chère collègue,

Nous avons le plaisir de vous informer que le comité de direction du REPAR a approuvé la recommandation du comité d'évaluation scientifique de soutenir votre demande dont le titre apparaît en rubrique.

Un budget de [redacted] $ a été approuvé

Nous joignons à cette lettre les évaluations de votre projet et les commentaires des évaluateurs et nous vous encourageons à les lire et à les prendre en compte.

Par la présente, nous vous prions de faire parvenir une copie de cet avis d'octroi à chacun de vos collègues. En recevant ce financement, vous prenez la responsabilité de soumettre un rapport financier officiel et un rapport scientifique dans les trois (3) mois suivant la fin du projet, c'est-à-dire au plus tard en décembre 2022. Tout membre d'une équipe qui n'aura pas déposé ces documents dans les délais prescrits se verra dans l'impossibilité d'obtenir un autre soutien du réseau tant que les documents ne seront pas remis.

Nous vous rappelons qu'aucune prolongation ne sera possible. Le FRQS nous demande d'appliquer de façon très stricte cette nouvelle règle concernant les prolongations de projet. Toute somme non dépensée sera retournée au REPAR.

De plus, vous vous engagez à :

- souscrire aux pratiques les plus rigoureuses en matière d'éthique de la recherche et d'intégrité scientifique,

- respecter les règles générales communes des Fonds de recherche du Québec,
- reconnaître la contribution financière du REPAR dans toute publication et tout document réalisés grâce à ce projet et informer le bureau de coordination du REPAR ;
- participer aux activités du REPAR.

À titre de chercheur principal, vous serez responsable de gérer ce projet en collaboration avec les codemandeurs. Veuillez noter que si un des codemandeurs a un statut d'étudiant, vous ne pouvez le rémunérer à même cette subvention.

Au nom des membres des comités de direction et scientifique du REPAR, je vous félicite et vous souhaite bon succès dans vos activités de recherche et dans la réalisation de vos travaux. Ceux-ci nous permettent d'enrichir le secteur des sciences de la réadaptation dans un contexte de réalisation d'activités pluridisciplinaires et multicentriques.

Veuillez agréer, Madame, l'expression de mes salutations distinguées.

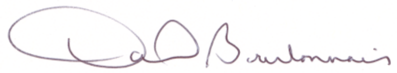

Daniel Bourbonnais
Directeur scientifique du REPAR

DB/cm

1.1 Recherche clinique - Grille d'évaluation demande complète

87pts

La compilation des notes des différents évaluateurs sera en rang. Donc, vous cotez en termes de notes mais ce sont l'ordre dans lequel nous classerons les demandes qui seront considérées. Ceci évitera qu'un évaluateur qui donne des notes plus hautes influence le résultat.

0/0pts

1. Originalité et pertinence en regard des objectifs du programme

19/20pts

A. Originalité et caractère innovant de la question de recherche

○ 7 ○ 8 ○ 9 ○ 10 ○ 11 ○ 12 ○ 13 ○ 14 ○ 15

B. Pertinence scientifique en regard des objectifs du programme

○ 0 ○ 1 ○ 2 ○ 3 ○ 4 ○ 5

Commentaire

Problématique pertinente est bien décrit. Le rationale est bien en ligne avec les objectifs. C'est une project originale aussi.

2. Qualité scientifique du projet

38/45pts

A. Justification du problème ou de la question de recherche

○ 5 ○ 5.5 ○ 6 ○ 6.5 ○ 7 ○ 7.5 ○ 8 ○ 8.5 ○ 9 ○ 9.5 ○ 10

B. Devis, population étudiée, recrutements des sujets, instruments de mesure, etc. clairement décrits

○ 10 ○ 11 ○ 12 ○ 13 ○ 14 ○ 15 ○ 16 ○ 17 ○ 18 ○ 19 ○ 20

C. Variables retenues, méthodes d'analyses bien décrites et appropriées

○ 7 ○ 8 ○ 9 ○ 10 ○ 11 ○ 12 ○ 13 ○ 14 ○ 15

Commentaire

La justification du problème est clair et convaincante. Le besoin est bien décrit. Les objectifs specifique est bien écrit, mais je questionnée si Obj 2 est vraiment un objective de recherche.

Le premier objectif (développement d'intervention) me semble à cet effet suffisant. De plus, la méthodologie associée à ce volet est appropriée.

Il me semble que le 2e obj manque des elements de recherche, et sera plus a mettre en place (e.g., Il manque des evaluation d'implantation pour examples, mais c'est pas le bon moment a faire un étude d'implantation quand même).

Le 3e objectif est un peut ambitieux (évaluation d’acceptabilité et feasibilité), mais sera un petite n et c’est bien montré dans le enchancier. L’evaluation d’acceptabilité est bien décrit, mais il manques des indicateurs de feasibilité. Le guide d’entrevue est base sur un bon theorie pour l’accpetabilité, mais il parlé pas de feasibailite. Le plan d’analysés est sound.

---

3. Retombées potentielles du projet

30/35pts

A. Potentiel des retombées par rapport à la pratique clinique en adaptation-réadaptation

○ 5 ○ 5.5 ○ 6 ○ 6.5 ○ 7 ○ 7.5 ○ 8 ○ 8.5 ◉ 9 ○ 9.5 ○ 10

B. Potentiel du projet pour l’obtention de subventions ultérieures auprès d’organismes de financement externes lors de concours réguliers

○ 5 ○ 5,5 ○ 6 ○ 6.5 ○ 7 ○ 7.5 ○ 8 ○ 8.5 ◉ 9 ○ 9.5 ○ 10

C. Qualité (pertinence et réalisme) du plan de transfert ou d’appropriation des connaissances

○ 7 ○ 8 ○ 9 ○ 10 ○ 11 ◉ 12 ○ 13 ○ 14 ○ 15

Commentaire

Une approche participative garantira que la voix des participants sera entendue pour assurer la contextualisation et la prise en compte de leurs besoins. Les résultats de cette étude éclaireront un projet plus large et le développement d'un programme de recherche, incluant l'utilisation d'approches de recherche participative pour cette population (important pour le programme 1.1). Le chercheur a une bonne avance pour une possibilité de financement supplémentaire et propose un travail pilote convaincant pour être compétitif pour un financement futur. Il faudra peut-être envisager des possibilités de financement moins importantes avant les IRSC pour obtenir un projet pilote plus convaincant. Le plan de TC est bien rédigé pour le public académique (i.e., les revues et les conférences), mais le plan général de TC incluant la participation des participants (i.e., la recherche-action participative pourrait être mieux décrite ... comment les participants seront-ils impliqués dans le TC?)

Commentaire général à propos de cette évaluation ou de la demande

1.1 Recherche clinique - Grille d'évaluation demande complète

88pts

La compilation des notes des différents évaluateurs sera en rang. Donc, vous cotez en termes de notes mais ce sont l'ordre dans lequel nous classerons les demandes qui seront considérées. Ceci évitera qu'un évaluateur qui donne des notes plus hautes influence le résultat.

0/0pts

---

1. Originalité et pertinence en regard des objectifs du programme

18/20pts

A. Originalité et caractère innovant de la question de recherche

○ 7 ○ 8 ○ 9 ○ 10 ○ 11 ○ 12 ◉ 13 ○ 14 ○ 15

B. Pertinence scientifique en regard des objectifs du programme

○ 0 ○ 1 ○ 2 ○ 3 ○ 4 ◉ 5

Commentaire

1. Originalité et pertinence:
Le projet est innovant puisqu'il ne semble pas y avoir d'intervention multidisciplinaire pour la reprise du travail chez les femmes avec un cancer du sein, et en lien avec l'utlisation de la méthode de co-design. Par contre, n'existe-t-il pas déjà des interventions de retour au travail pour d'autres populations cliniques sur lesquelles se baser (ex.: post-TCC, santé mentale)? Le projet répond à la mission du REPAR et est en lien avec les projets structurants PS-V2 et PS-C2, quoique ce n'est pas explicite dans le texte.

---

2. Qualité scientifique du projet

37/45pts

A. Justification du problème ou de la question de recherche

○ 5 ○ 5.5 ○ 6 ○ 6.5 ○ 7 ○ 7.5 ○ 8 ○ 8.5 ◉ 9 ○ 9.5 ○ 10

B. Devis, population étudiée, recrutements des sujets, instruments de mesure, etc. clairement décrits

○ 10 ○ 11 ○ 12 ○ 13 ○ 14 ○ 15 ◉ 16 ○ 17 ○ 18 ○ 19 ○ 20

C. Variables retenues, méthodes d'analyses bien décrites et appropriées

○ 7 ○ 8 ○ 9 ○ 10 ○ 11 ◉ 12 ○ 13 ○ 14 ○ 15

Commentaire

2. Qualité scientifique du projet:
La problématique est très bien présentée, appuyée sur des modèles théoriques, et la pertinence du projet est justifiée. Il aurait été intéressant de présenter les données disponibles sur les déterminants du retour au travail (ex.: type d'emploi, pérennité, charge, etc.). Le recrutement pour l'objectif 1 est un peu flou (6-10 personnes, provenance/répartition des groupes de parties prenantes - s'il s'agit d'un projet de faisabilité, est-ce préférable d'avoir des personnes provenant d'un seul milieu clinique/type d'emploi?). Par ailleurs, le milieu pour les objectifs 2-

3 ne semble pas identifié, ce qui est crucial puisque cela exigera un changement dans la pratique de ce milieu. Les analyses qualitatives sont bien décrites et appropriées, mais il manque d'information sur le traitement des données récoltées à l'objectif 1.

3. Retombées potentielles du projet

33/35pts

A. Potentiel des retombées par rapport à la pratique clinique en adaptation-réadaptation

○ 5 ○ 5.5 ○ 6 ○ 6.5 ○ 7 ○ 7.5 ○ 8 ○ 8.5 ○ 9 ○ 9.5 ○ 10

B. Potentiel du projet pour l'obtention de subventions ultérieures auprès d'organismes de financement externes lors de concours réguliers

○ 5 ○ 5,5 ○ 6 ○ 6.5 ○ 7 ○ 7.5 ○ 8 ○ 8.5 ○ 9 ○ 9.5 ○ 10

C. Qualité (pertinence et réalisme) du plan de transfert ou d'appropriation des connaissances

○ 7 ○ 8 ○ 9 ○ 10 ○ 11 ○ 12 ○ 13 ○ 14 ○ 15

Commentaire

3. Retombées:
Les retombées du projet sont clairement avancées, et le texte présente de façon claire les étapes futures d'obtention de financement additionnel. Le plan de transfert de connaissances est classique, mais aurait pu être bonifié en lien avec l'utilisation d'approche participative/co-design.

Commentaire général à propos de cette évaluation ou de la demande

1.1 Recherche clinique - Grille d'évaluation demande complète

95pts

La compilation des notes des différents évaluateurs sera en rang. Donc, vous cotez en termes de notes mais ce sont l'ordre dans lequel nous classerons les demandes qui seront considérées. Ceci évitera qu'un évaluateur qui donne des notes plus hautes influence le résultat.

0/0pts

1. Originalité et pertinence en regard des objectifs du programme

18/20pts

A. Originalité et caractère innovant de la question de recherche

○ 7 ○ 8 ○ 9 ○ 10 ○ 11 ○ 12 ○ 13 ○ 14 ○ 15

B. Pertinence scientifique en regard des objectifs du programme

○ 0 ○ 1 ○ 2 ○ 3 ○ 4 ○ 5

Commentaire

Le rationnel est notamment axé sur les besoins de la personne, en phase avec les approches promeut actuellement par les IRSC (approche centrée sur le patient) et FRQ-S (approche Laboratoire Vivant). Bien que la proposition comprenne quelques autocitations, celles-ci tendent à démontrer la solidité et la spécificité de son créneau de recherche.

Équipe intersectorielle, au de-là de la réadaptation (gestion et design). L'accent est mis sur la réadaptation de première ligne. Description de l'expertise et du rôle de chacun des membres, incluant les collaborateurs cliniciens.

- Il aurait été intéressant d'inclure un comité de pilotage sur lequel siège un patient-partenaire.
- Il serait souhaitable que la chercheuse démontre la complémentarité de ce projet avec la subvention FRQ-Jeune chercheuse.

2. Qualité scientifique du projet

44/45pts

A. Justification du problème ou de la question de recherche

○ 5 ○ 5.5 ○ 6 ○ 6.5 ○ 7 ○ 7.5 ○ 8 ○ 8.5 ○ 9 ○ 9.5 ○ 10

B. Devis, population étudiée, recrutements des sujets, instruments de mesure, etc. clairement décrits

○ 10 ○ 11 ○ 12 ○ 13 ○ 14 ○ 15 ○ 16 ○ 17 ○ 18 ○ 19 ○ 20

C. Variables retenues, méthodes d'analyses bien décrites et appropriées

○ 7 ○ 8 ○ 9 ○ 10 ○ 11 ○ 12 ○ 13 ○ 14 ○ 15

Commentaire

La pertinence sociale et scientifique de l'étude est très solide et décrite avec clarté – la problématique est bien

positionnée, alors que la recension des écrits fort bien documentée. La collecte de données et l’analyse reposent sur des modèles pertinents et récents, lesquels sont exposés clairement. Démontre une connaissance approfondie de l’approche co-design.

- Objectifs clairs et ambitieux, mais la chercheuse reconnaît les limites potentielles de l’approche proposée et évoque des moyens pour les atténuer. Penser à une collecte de données transversale des processus pour les deux premiers objectifs?

- Il aurait été intéressant d’exposer les éléments à considérer / limites potentielles liées à l’utilisation de Zoom pour les ateliers de co-design (biais de sélection dans la littératie numérique / fatigue cognitive) et les stratégies à déployer pour s’assurer d’une représentativité des patients/participants.

---

3. Retombées potentielles du projet

33/35pts

A. Potentiel des retombées par rapport à la pratique clinique en adaptation-réadaptation

○ 5 ○ 5.5 ○ 6 ○ 6.5 ○ 7 ○ 7.5 ○ 8 ○ 8.5 ◉ 9 ○ 9.5 ○ 10

B. Potentiel du projet pour l’obtention de subventions ultérieures auprès d’organismes de financement externes lors de concours réguliers

○ 5 ○ 5,5 ○ 6 ○ 6.5 ○ 7 ○ 7.5 ○ 8 ○ 8.5 ○ 9 ○ 9.5 ◉ 10

C. Qualité (pertinence et réalisme) du plan de transfert ou d’appropriation des connaissances

○ 7 ○ 8 ○ 9 ○ 10 ○ 11 ○ 12 ○ 13 ◉ 14 ○ 15

Commentaire

- Effet levier intéressant du projet.
- Stratégies de transfert de connaissances originales et pertinentes (diffusion du cahier d’intervention à la fin du projet).
- Budget pertinent. Essentiellement alloué au personnel de recherche, ce qui est conforme aux dépenses anticipées. Compensation salariale pour 2 heures (100$) conformes aux règles de compensation pour les patients partenaires.

o Réfléchir si besoins de productions audiovideo ou de matériel pour les séances de co-design ou liée à la diffusion de connaissances (matériel web).

o Identifier les activités préconisées lors de la séance pour coconstruire l'intervention et arriver à un livrable en 2 heures.

Commentaire général à propos de cette évaluation ou de la demande

Excellente demande - scientifiquement solide et pertinente. Il importe toutefois de vérifier la présence de chevauchements entre ce projet et le financement déjà obtenu pour soutenir sa programmation de recherche (ex. FRQ).
